# Emerging roles of eraser enzymes in the dynamic control of protein ADP-ribosylation

**DOI:** 10.1038/s41467-019-08859-x

**Published:** 2019-03-12

**Authors:** Julia O’Sullivan, Maria Tedim Ferreira, Jean-Philippe Gagné, Ajit K. Sharma, Michael J. Hendzel, Jean-Yves Masson, Guy G. Poirier

**Affiliations:** 1 Genome Stability Laboratory, Centre de Recherche du Centre Hospitalier Universitaire de Québec-Université Laval, HDQ Pavilion, Oncology Division, Québec, G1R 2J6 Canada; 2 Département de Biologie Moléculaire, Biochimie Médicale et Pathologie, Faculté de Médecine, Université Laval, Québec, G1V 0A6 Canada; 3 Centre de Recherche du Centre Hospitalier Universitaire de Québec-Université Laval, CHUL Pavilion, Oncology division, Québec, G1V 4G2 Canada; 4 Department of Oncology, Faculty of Medicine and Dentistry, University of Alberta, Edmonton, T6G 1Z2 Canada; 5 Department of Cell Biology, Faculty of Medicine and Dentistry, University of Alberta, Edmonton, T6G 2H7 Canada; 6 Centre de Recherche sur le Cancer de l’Université Laval, Québec, G1R 3S3 Canada

## Abstract

Protein ADP-ribosylation is essential for the regulation of several cellular pathways, enabling dynamic responses to diverse pathophysiological conditions. It is modulated through a dynamic interplay between ADP-ribose readers, writers and erasers. While ADP-ribose synthesis has been studied and reviewed extensively, ADP-ribose processing by erasing enzymes has received comparably less attention. However, major progress in the mass spectrometric identification of ADP-ribosylated residues and the biochemical characterization of ADP-ribose erasers has substantially expanded our knowledge of ADP-ribosylation dynamics. Herein, we describe recent insights into the biology of ADP-ribose erasers and discuss the intricately orchestrated cellular processes to switch off ADP-ribose-dependent mechanisms.

## Abstract (web summary)

ADP-ribose erasing enzymes are increasingly recognized as critical regulators of protein ADP-ribosylation dynamics in living systems. Here, the authors review recent advances in the discovery and characterization of ADP-ribose erasers and discuss their role within the cellular ADP-ribosylation machinery.

## Introduction

Reversible post-translational modifications (PTMs) contribute to the dynamic regulation of the proteome through a diversified repertoire of functions. Protein ADP-ribosylation has emerged as a complex, dynamic, and reversible PTM system within which fundamental components work antagonistically to fine tune and tightly regulate protein behavior^1^. Similar to other transient biological processes, the ADP-ribosylation turnover relies on synthesis and degradation mechanisms^2,3^. The enzymes that perform these functions can essentially be described as writers and erasers, a nomenclature borrowed from the classification of proteins involved in epigenetic regulation. ADP-ribose writers are collectively referred to as ADP-ribose transferases (ARTs), a family of proteins with mono- or poly(ADP-ribose) transferase activities. These enzymes, especially the promising drug target poly(ADP-ribose) polymerase-1 (PARP-1), have been intensely studied by the ADP-ribosylation community for many years. More recently, attention has shifted towards the biological roles of ADP-ribose erasers, stimulated by the identification of a variety of ADP-ribose degrading enzymes with different substrate specificities. These recent findings have profoundly changed the prevailing view that ADP-ribose erasing depends almost solely on poly(ADP-ribose) glycohydrolase (PARG) activity.

**Fig. 1:**
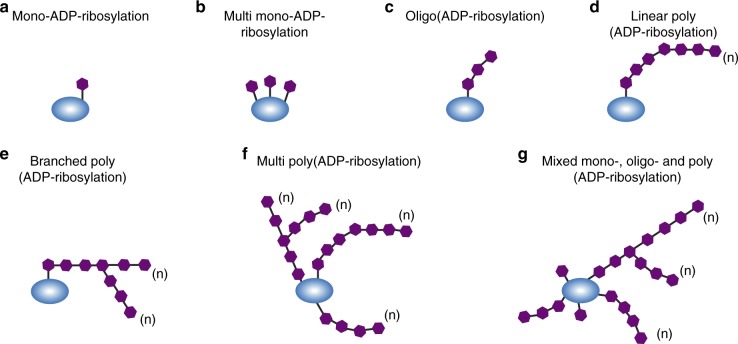
Possible patterns of ADP-ribosylation on target proteins. **a** Mono-ADP-ribosylation; a single ADP-ribose molecule is attached to the protein. **b** Multi mono-ADP-ribosylation; multiple single ADP-ribose units are bound along the protein. **c** Oligo(ADP-ribosylation); short linear chains of ADP-ribose are transferred to the protein. **d** Linear poly(ADP-ribosylation); ADP-ribose moieties forming a long linear chain up to 200 units in length. **e** Branched poly(ADP-ribosylation); complex molecules composed of large and branched polymers of ADP-ribose. **f** Multi poly(ADP-ribosylation); multiple PAR chains either linear or branched on the same protein. **g** Mixed ADP-ribosylation; a mixture of the previously described ADP-ribose patterns on the same protein, generated either by the combined action of MAR- and PAR transferases or by the degradative action of erasers

ADP-ribosylation—in its strictest sense—refers to the enzymatic addition of an ADP-ribose molecule to a target substrate. The transferrable ADP-ribosyl units are typically derived from NAD^+^ through the cleavage of the nicotinamide-ribosyl bond. Therefore, ADP-ribosylation reactions generally depend on NADase activity. A fundamental distinction exists between mono-ADP-ribosylation (MARylation), i.e., the transfer of a single ADP-ribose monomer, and poly(ADP-ribosylation) (PARylation), which involves the biosynthesis of elongated ADP-ribose polymers (Fig. 1). PAR polymers form nucleic acid-like polyanion structures that can serve as a docking site for a variety of reader domains (reviewed in ref. ^4^). MARylation can impact protein activity, stability, substrate specificity, folding, or localization. For instance, substrates of the bacterial MAR transferases can undergo substantial structural rearrangements that profoundly modify host cell physiology and promote cellular intoxication^5^. The functional divergence between MARylating and PARylating enzymes is consistent with a biological system that involves multiple layers of antagonizing activities. This concept is supported by a rapidly expanding repertoire of ADP-ribose-degrading enzymes, suggesting that MAR and PAR modifications are continuously transferred to, and removed from, substrates by an antagonizing set of enzymes.

This review will first focus on PARG and the more recently characterized enzymes that can reverse ADP-ribosylation. Subsequently, we will discuss the biochemical methods used to detect ADP-ribosylation turnover, and expand on the regulation of ADP-ribosylation through combinatorial selective erasing mechanisms. We will conclude by discussing the therapeutic target potential of ADP-ribose erasers, focusing on the use of PARG inhibitors in synthetic lethal approaches against cancer.

## Enzymes involved in the removal of ADP-ribosylation

Recent advances in defining ADP-ribose metabolism suggest that the balance between ADP-ribose writers and erasers is crucial for the coordination of multiple cellular response pathways^6^. This view is supported by the identification of a growing number of proteins implicated in writing, reading, and erasing the ADP-ribosylation modifications. Although a synthesis and degradation duality is inherent to transient PTMs, specialized erasers might occupy different catalytic niches to provide a functional and temporal reversibility of the reaction and for the recycling of ADP-ribosylated substrates. The inability of PARG—the main dePARylating enzyme—to remove MARylation marks^7,8^, and its limited processivity on short PAR polymers, leaves room for the involvement of other erasers (Table 1). A complete reversal of MARylation is performed in human cells by amino-acid-specific ADP-ribose-acceptor hydrolases, such as the macrodomain-containing proteins MacroD1 and MacroD2, the terminal ADP-ribose protein glycohydrolase 1 (TARG1), and the ADP-ribose hydrolase (ARH) family members ARH1 and ARH3. Moreover, several phosphodiesterases have been shown to possess ADP-ribose processing activity. In this section, we provide an overview of these different ADP-ribose erasing enzymes.

**Table 1:** Human ADP-ribose erasers

Eraser	Classification	Substrate	Targeted bond	ADP-ribosylation reversal	Protein adduct	Amino acid selectivity	References
PARG	Macrodomain	PAR	O-glycosidic	Partial	ADP-ribose	Linkage-independent	^23, 80^
MacroD1	Macrodomain	MAR	Carboxyl ester	Complete	None	D/E	^84^
MacroD2	Macrodomain	MAR	Carboxyl ester	Complete	None	D/E	^84^
TARG1	Macrodomain	MAR/PAR	Carboxyl ester	Complete	None	D/E	^58^
ARH1	ARH fold	MAR	N-glycosidic	Complete	None	R	^48^
ARH3	ARH fold	MAR/PAR	O-glycosidic	Complete	None	S	^46, 48^
NUDT9	NUDIX	PAR	Phosphodiester	Partial	Phosphoribose	Linkage-independent	^65^
NUDT16	NUDIX	MAR/PAR	Phosphodiester	Partial	Phosphoribose	Linkage-independent	^65, 66^
ENPP1	ENPP (PDNP)	MAR/PAR	Phosphodiester	Partial	Phosphoribose	Linkage-independent	^67^

### Poly(ADP-ribose) glycohydrolase (PARG)

Although a role of MARylation in response to genotoxic stress has become better established recently (reviewed in ref. ^9^), only PARylation occurs in conjunction with a substantial decrease of intracellular NAD^+^ concentrations when extensive DNA damage is encountered. Globally, PARylation processes account for a large proportion of the ART activity in cells. Therefore, dePARylation can be viewed as the predominant erasing activity.

**Fig. 2:**
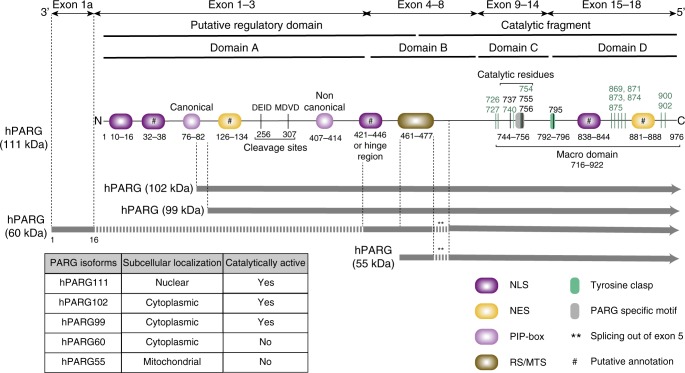
Schematic representation of human PARG and its isoforms. Human PARG originates from a 3198 bp mRNA sequence with a single 2931 bp open reading frame (ORF). The ORF contains 18 exons and encodes a protein of 976 amino acids with a molecular weight of 111.1 kDa. This mRNA undergoes alternative splicing to produce different PARG isoforms. Five human PARG transcripts have been identified. Full-length human PARG (hPARG111) contains an N-terminal regulatory domain and a C-terminal catalytic domain that is essentially a macrodomain fold. hPARG102 and hPARG99 are translated from the start codons located in exon 2 and exon 3, respectively. hPARG60 results from alternative splicing that connects exon 1a, exon 4, and exons 6–18. Because of the usage of a facultative exon (exon 1a), hPARG60 has an alternative N-terminal protein sequence of 16 amino acids that is unique to this isoform. hPARG55 is produced from the initiation of translation at the start codon located in exon 4. Exon 5 is spliced out in both hPARG60 and hPARG55 isoforms. PARG can be sub-classified into four different domains. Domain A, which includes exons 1–3, forms the majority of the putative regulatory domain. This region contains two caspase-3 cleavage sites at amino acid position 256 (DEID) and 307 (MDVD). An uncharacterized nuclear localization signal (NLS) overlaps with a hinge region between the putative regulatory domain and the catalytic fragment. Furthermore, PARG comprises domain B (exons 4–8), domain C (exons 9–15) and domain D (exons 15–18). The latter two domains form the base of the macrodomain fold and contain the catalytic pocket and ligand binding sites. The catalytic residues (Asp737, Glu755, and Glu756) and Tyr795, which interacts with PARG inhibitor ADP-HPD are indicated as black lines. Residues colored in green have been implicated in the binding of ADP-ribose. Furthermore, colored boxes denote PARG-specific motif (GGG-X_6–8_-QEE), regulatory segment/mitochondrial targeting sequence (RS/MTS), Tyrosine Clasp structural motif, PCNA-interacting protein (PIP) motif, and nuclear export signal (NES)

PARG is the major dePARylating enzyme, and is primarily responsible for hydrolyzing the glycosidic linkages between ADP-ribose units of PAR polymers to generate free ADP-ribose monomers. Only a single *PARG* gene has been identified in mammals and its sequence is highly conserved^10^. *PARG* homologs are detected in a wide range of eukaryotes with the exception of budding yeast. The human *PARG* gene encodes for multiple variants produced by alternative splicing of a unique mRNA^11,12^. The characterization of *PARG* expression products and the apparent molecular weight heterogeneity of PARG have been reviewed elsewhere^13^. PARG is a modular protein with a four domain architecture^10^ (Fig. 2). Domain A spans exons 1–3 and forms a predicted N-terminal intrinsically disordered regulatory domain^14^. Domain B (exons 4–8) connects the N-terminal region to the catalytic domain through a hinge region^11^ and contains a regulatory segment^15^. Domain C (exons 9–14) contains the catalytic active site and the PARG signature motif^16^. Domains C and the C-terminal domain D (exons 15–18) form the PARG macrodomain^7,17^. A number of nuclear export signals (NES) and nuclear localization signals (NLS) are distributed throughout the PARG sequence.

The expression of a variety of *PARG* splice variants with different localizations enables functional specialization^11,18^. In human cells, major isoforms include a full length 111 kDa PARG enzyme and splice variants that generate proteins of 102 and 99 kDa (Fig. 2). While full length PARG is mostly nuclear and accounts for a minor fraction of global cellular activity, the smaller isoforms localize primarily to the cytoplasm with a perinuclear distribution, and seem to be responsible for most of the PAR processing activity^19^. Therefore, nuclear and cytoplasmic compartmentalization, and the shuttling of PARG isoforms between the nucleus and cytoplasm have been proposed as a mechanism to regulate cellular PAR levels^18,20^. PARG mRNA also undergoes additional alternative splicing that generates small isoforms of 55 and 60 kDa. Both hPARG55 and hPARG60 isoforms have been found to be catalytically inactive due to the absence of exon 5-encoded amino-acids^21^ (Fig. 2). Therefore, these small human PARG isoforms are not involved in general PAR turnover in cells.

Human PARG is a constitutively active, low abundance enzyme that possesses both exoglycosidase and endoglycosidase activities. PARG mainly functions as an exoglycosidase, sequentially digesting glycosidic linkages from the protein-distal end of the polymer similar to carbohydrate glycosyl hydrolases^22^. This processivity improves the catalytic activity of PARG but is strongly chain-length dependent^23^. On the other hand, it has been estimated that ~20% of PARG depolymerization activity can be accounted for by in-chain endoglycosidic degradation^24^. The fact that PARG has two mechanisms of action with different degradation kinetic parameters and PAR structure-affinity might be an overlooked characteristic in the complex and intricate interplay between ADP-ribose readers and erasers, as will be discussed further below.

The importance of the catalytic activity of PARG became clear with the observation that *PARG*^−/−^ mice were embryonic lethal^25^ and that PARG-depleted cells are hypersensitive to genotoxic insults^26,27^. This is accompanied by PAR accumulation and early apoptosis, suggesting that efficient PARG-mediated PAR turnover is required for the recovery from DNA damage. PARG has also been shown to be necessary to prevent massive PAR production upon prolonged replicative stress^28^. Schreiber and colleagues demonstrated that PARG deficiency delays cellular recovery from persistent replication stress, triggered by prolonged hydroxyurea treatment^28^. These blocked cells display high PAR levels, which negatively impacts RPA foci formation and its association with single-stranded DNA (ssDNA). The prevention of RPA loading eventually leads to increasing areas of uncovered ssDNA, which then transform into DNA double-strand breaks (DSBs), resulting in the formation of more PAR. Ultimately, this amplification loop promotes apoptosis and/or necrotic cell death in proliferating cell populations. These observations are in agreement with the finding that PARG localizes to replication foci throughout S-phase and interacts with the replication protein PCNA^29,30^. Furthermore, they complement an earlier report that PARG-deficient cells treated with DNA alkylating agents have an increase in S-phase arrest together with high levels of the DSB marker γH2AX^31^. Correspondingly, the Lopes group showed that PARG inactivation affects the progression of all replication forks and alters the molecular architecture of a significant fraction of replication intermediates^32^. These results provided mechanistic insight into the essential role of PARG in cell growth and development, in line with the observed embryonic lethality of *PARG*^−/−^ mice^33^.

Another important role of PARG during the DNA damage response is to maintain stable levels of PAR and to recycle highly automodified PARP-1. The stabilization of PAR levels is crucial for protecting the cell against parthanatos, a caspase-independent PAR-mediated type of cell death^34^. Parthanatos is triggered by the release of the apoptosis-inducing factor (AIF) from the mitochondria to the nucleus^35,36^. Once translocated to the chromatin environment, AIF leads to large-scale DNA fragmentation and chromatin condensation, which is followed by cell death^37^. Depletion of PARG has been shown to be protective against oxidative stress-induced parthanatos by preventing the release of AIF from the mitochondria^38^.

Lastly, PARG has also been implicated in telomere maintenance. PARG is capable of negatively regulating the access to telomeric DNA by reversing ADP-ribosylation of the telomeric-specific protein TRF1, contributing to the regulation of telomere repair and replication^39,40^. Overall, these examples show that the dynamic equilibria established between PARP-1 and PARG activities, and therefore PAR levels, are key for controlling cell fate, suggesting that PAR erasers are as important as PAR writers for cellular homeostasis.

### ADP-ribose hydrolases (ARHs)

ADP-ribose conjugation was first described as a PTM catalyzed by bacterial ADP-ribosylating exotoxins (bAREs)^41^. Bacterial MAR transferases (MARTs) have related genes in humans whose extracellular expression makes them irrelevant or inoperative with respect to intracellular ADP-ribose-mediated pathways^42^. In human cells, intracellular protein MARylation is performed by members of the ADP-ribosyl transferases diphtheria toxin-like proteins (ARTDs). Formerly classified as PARPs^43^, the 17 members of the ARTD family in human were renamed according to a systematic nomenclature that better reflects their structural features and catalytic properties^44^. There are currently 11 members of the human ARTD family characterized as MARTs, typically renamed after the type of ADP-ribose molecule (i.e., MAR) they transfer onto themselves or target substrates^45,46^.

**Fig. 3:**
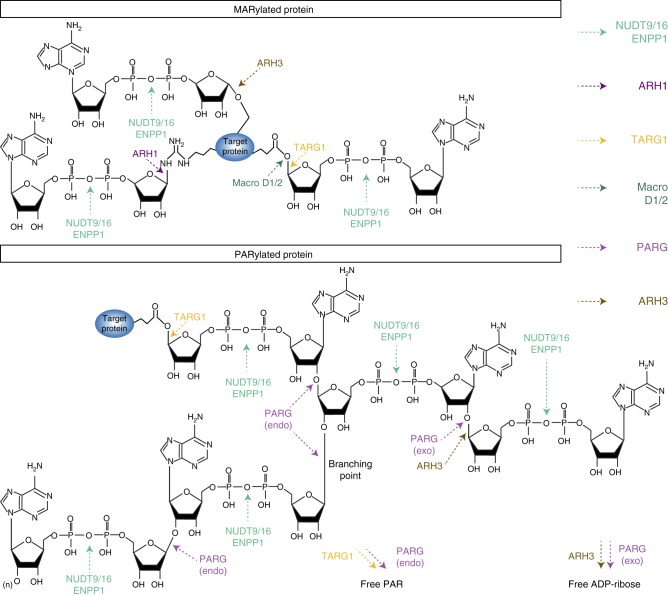
Reversal of protein ADP-ribosylation by MAR and PAR erasers. The diagrams represent MARylated (upper panel) and PARylated proteins (lower panel) with bond-specific chemical cleavage sites for each eraser. A subgroup of erasers that comprises MacroD1, MacroD2, and ARH1 are MAR-specific erasers involved in the removal of single ADP-ribose adducts. MacroD1 and MacroD2 are macrodomain-containing enzymes that release ADP-ribose from ADP-ribosylated acidic residues (aspartate and glutamate). ARH1 is currently the only known MAR hydrolase that specifically removes MAR from arginine residues. A second subgroup that includes TARG1, ARH3, NUDT9, NUDT16, and ENPP1 can target both MAR and PAR modifications. The TARG1 macroprotein hydrolyzes glutamate-ADP-ribose bonds and releases ADP-ribose from MARylated proteins. TARG1 has also the unique ability to remove entire PAR chains from acidic residues of PARylated proteins. ARH3 is limited to exoglycosidic activity toward PAR chains and releases free ADP-ribose. In addition, it possesses MAR hydrolase activity specifically targeting the O-linked ADP-ribosylation. NUDT9 and NUDT16 have nucleoside diphosphate-linked moiety-X (NUDIX) domains, which cleave pyrophosphate bonds and release phospho-ribosyl-AMP from PAR chains or AMP from MARylated proteins as major reaction products. ENPP1 is a pyrophosphatase lacking a NUDIX domain but with the capability of digesting PAR and MAR modifications similar to NUDIX enzymes. PARG is the main PAR-degrading enzyme but shows no activity towards MARylated proteins. Human PARG is unable to cleave the proximal ADP-ribose groups from a modified protein but possesses exo- and endoglycosidic activities to hydrolyze the glycosidic bonds between ribose units of PAR. The exoglycosidic activity of PARG generates free ADP-ribose from the processive degradation of PAR from the distal to the proximal end while its in-chain cleavage activity (endoglycosidic) produces protein-free PAR. The endoglycosidic degradation of PAR by of PARG is also responsible for the hydrolysis of the branching points formed when non-adenine riboses are linked together (branching point)

The ADP-ribose hydrolase (ARH) family consists of three related proteins^47^. While ARH2 substrates are yet to be discovered, ARH1 is a highly active ADP-ribosyl-arginine hydrolase^48^ and ARH3 is an ADP-ribosyl-serine hydrolase^49^ (Table 1 and Fig. 3). Mice that lack ARH1 are more sensitive to cholera toxin^50^ and tumor-prone, having increased incidences of adenocarcinoma, lymphoma, and metastases^51^. ARH3-deficient mouse embryonic fibroblasts show increased steady-state abundance of serine-ADP-ribosylation in vivo^52^ and DNA damage-induced serine-ADP-ribosylation is efficiently reversed by ARH3^49^. In contrast to ARH1, ARH3 also possesses activity toward the O-glycosidic bond of PAR, similar to the exoglycosidic activity of PARG^42^. However, ARH3 does not rescue *Drosophila* or mouse genetic knockouts of PARG from cell death or PAR accumulation^25,53^, suggesting that it cannot compensate for the loss of PARG. Owing to its abundance in the cytoplasm, ARH3 participates in a second stage of PAR hydrolysis following the release of free PAR polymer branches by other erasers. This may help lower the cytoplasmic PAR levels, ultimately preventing mitochondria-dependant apoptotic pathways such as parthanatos^54^.

While ARHs only erase arginine- and serine-MARylation and macrodomain-containing enzymes specifically target aspartate- and glutamate-MARylation (see next section), ARTDs have been shown to mediate ADP-ribosylation on a wide range of amino-acid residues^55,56^ This apparent discrepancy will be discussed later on in this article.

### Macrodomain-containing ADP-ribose erasers

The macrodomain fold is an evolutionarily conserved, compact globular-shaped structure of ~25 kDa present throughout all of the biological kingdoms^57^. It can be found as a stand-alone module or integrated into multi-domain proteins. The macrodomain was the first characterized ADP-ribose-binding module. It can bind terminal ADP-ribose structures with nanomolar affinity^58^. There is functional diversity related to structural variation in the macrodomain protein family. A subgroup of macrodomains lacks the ability to bind ADP-ribose while others acquired glycosidic activity involved in ADP-ribosylation reversal^4^. There are ten human macrodomain-coding genes: the histone H2A variants Macro H2A.1 and Macro H2A.2; MacroD1, D2, and D3; TARG1; the chromodomain-helicase-DNA-binding protein 1-like (CHD1L) and the macrodomain-containing ARTDs 7-8-9 (formerly named PARPs 15-14-9). Among these, MacroD1, MacroD2, and TARG1 were classified as ADP-ribose erasers because of their ADP-ribose hydrolase activities. MacroD1 and MacroD2 cleave the chemical link between MAR and an acceptor protein while TARG1 presents the unique capability of cleaving both MARylated and PARylated side chains of aspartate and glutamate residues^59^ (Fig. 3).

The role of TARG1 in PAR turnover remains elusive but a PARylation-dependent relocation of TARG1 to the nucleoplasm has been observed^60^ in addition to its recruitment to DNA lesions in a PAR-dependent fashion^59^. The catalytic domain of TARG1 is different from PARG but resembles the OGG1 DNA glycosylase^59^ and directly targets the carboxyl ester ADP-ribose linkages to remove the modification from its substrate. The ability of TARG1 to remove whole PAR chains from the substrate most proximal attachment point is unique among the known erasers, adding another putative regulatory layer to PAR cellular functions. For years, only PARG was known to generate protein-free ADP-ribose polymers as a consequence of its endoglycosidic activity^23^ (Fig. 3), which becomes the major mode of action when robust PARP activation (i.e., strong genotoxic insults) leads to the synthesis of large and branched PAR^61^. Although this idea has not been fully evaluated, TARG1-mediated production of protein-free PAR might be involved in parthanatos^59^.

Similarly to TARG1, the mono-ADP-ribose hydrolase activities of MacroD1 and MacroD2 are also selectively directed toward ester bonds established by ADP-ribosylated aspartate and glutamate residues, although with different catalytic modes (reviewed in ref. ^62^). Current experimental data suggest that ester-type ADP-ribose bonds in protein substrates are specific targets of the macrodomain erasers. This activity could play a regulatory role in vivo as MacroD2, for example, has been implicated in the recycling of automodified PARP-1^63^. The removal of the autoinhibitory MAR moieties from PARP-1 by MacroD2 has been suggested to explain the accumulation of MARylated PARP-1 in the context of MacroD2 gene deletion in human colorectal cancer cells^64^. The underlying MacroD2-dependent PARP-1 recycling model proposed by Sakthianandeswaren et al. involves a biphasic erasing of PARP-1 automodification, which implicates PARG as the primary PAR trimming enzyme responsible for the generation of MAR adducts that can subsequently be targeted by MacroD2^64^.

As mentioned above, PARG is a member of the macrodomain eraser family, although there is no similarity between the amino-acid sequence of PARG and other macrodomain-containing proteins. However, there is a close structural and evolutionary relationship between macrodomains and PARG^17^, and its catalytic center is essentially a macrodomain fold^7,17^.

### Phosphodiester ADP-ribose hydrolases

Homopolymers of PAR are composed of successive ADP-ribose moieties linked together by alternating phosphodiester and O-glycosidic linkages (Fig. 3). The phosphodiester bond is also central to the ADP-ribose monomer itself as it links the adenosine structure to the ribose. The activity of snake venom phosphodiesterases was instrumental in the elucidation of PAR structure in the early studies of PARylation, as it was used to determine chain length and PAR branching frequency^65^. Only recently, a role of phosphodiesterases in the reversal of ADP-ribosylation has been proposed, following the discovery of a group of ADP-ribose processing phosphodiesterases that includes NUDIX (nucleoside diphosphates linked to moiety-X) superfamily members NUDT9 and NUDT16 as well as ectonucleotide pyrophosphatase/phosphodiesterase 1 (ENPP1)^66–68^. These erasers target the phosphodiester bound in ADP-ribose moieties. Therefore, their activity is independent of the type of ADP-ribose linkage established with the substrate protein. However, these enzymes should be classified as partial erasers since they leave a phosphoribose remnant attached to the target protein (Fig. 3 and Table 1). It is still unclear whether these phosphoribose remnants are correlated with specific biological outcomes but a pathological accumulation of phosphoribose on glutamate residues has been described^69^. Furthermore, the phosphodiesterase-catalyzed removal of the distal adenine in PAR ploymers through cleavage of the terminal AMP likely prevents digestion by PARG, as it was observed with etheno-PAR, a derivatized PAR with modified adenine moieties^70^.

In vivo, NUDIX hydrolases seem to fulfil ‘housekeeping’ functions, facilitating the detoxification of potentially deleterious endogenous metabolites^71^. Furthermore, they have been proposed to be involved in replenishing the cellular AMP pool from ADP-ribose monomer products of PARG/ARH3-mediated PAR depolymerization. This metabolic response is consistent with the AMP-dependent mitochondrial energy failure observed following DNA damage and PARP-1 activation^72^. The accumulation of PAR-derived AMP has also been implicated in the modulation of mTOR signalling through AMPK activation^73^. These examples show that ADP-ribose erasing reactions can have diverse effects on metabolism by generating free ADP-ribose monomers and related molecules such as AMP.

Interestingly, the hydrolase activity of a third NUDIX, NUDT5, diverges from the other ADP-ribose-processing NUDIX hydrolases because it cannot hydrolyze protein-conjugated ADP-ribose. However, NUDT5 generates ATP from free ADP-ribose and pyrophosphate in a recycling-like process to quickly replenish nuclear ATP levels^74^. While NUDT5 cannot be classified as an ADP-ribose eraser per se because of its inability to remove protein ADP-ribosylation, it certainly deserves attention as it can influence the level of energetic substrates following PAR catabolism.

The extracellular ENPP1 phosphodiesterase, which lacks a NUDIX and a macrodomain, is yet to be characterized regarding its involvement in ADP-ribose processing. ENPP1 shows considerable phosphodiesterase activity in vitro against MAR and PAR, exceeding that observed for NUDT16 in a cell-free system^68^. The high conversion rate of ADP-ribosylation modifications to phosphoribose adducts by ENPP1 has been suggested as a key feature for the generation of phosphoribose signatures for analysis by liquid chromatography-mass spectrometry (LC-MS)^68^.

## Detection and evaluation of MAR and PAR erasing activities

The emergence of a variety of new players that modulate ADP-ribose catabolism underscore the urgent need for methods capable of rapidly measuring erasing activities. Historically, most assays were developed to measure the disappearance of PAR as a consequence of PARG glycosidic activity. Usually based on residual PAR precipitation assays, these methods give rise to inconsistencies when monitoring PARG activity^75^. Based on this observation, a thin-layer chromatography (TLC)-based strategy coupled to a radiolabed PAR substrate was developed to monitor ADP-ribose accumulation rather than substrate disappearance^75^. This TLC method has been used successfully to measure PARG activity in cell extracts and tissues^76^, characterize site-directed mutants^77^ and to evaluate the inhibitory strength of small molecules^78,79^. Later, the conversion of the reaction product, the monomeric ADP-ribose, into a quantifiable fluorophore has been reported as a nonradiometric and high-throughput assay for PARG activity^80^.

TLC assays are inadequate to demonstrate the contribution of individual PARG isoforms or additional PAR-degrading enzymes to global PAR erasing activity in cells. In this respect, PAR zymograms were developed to detect alternative catabolic activity against PAR in complex samples. Zymograms are essentially composed of radiolabeled automodified PARP-1 co-polymerized with a polyacrylamide gel. Following renaturation, digested regions can be visualized on the autoradiogram. Although protein renaturation and in-gel activity constraints the applicability of this strategy, zymography proved to be an effective and sensitive method to detect PAR hydrolysis by PARG^75^. However, no significant additional PAR erasing activity has been detected in most cell extracts using this approach, contributing to the long held belief that PARG was solely responsible for mediating PAR degradation in cells.

Consistent measurements of PARG activity under standardized conditions are hindered by the absence of a well-defined substrate (i.e., of defined length and branching frequency). Additionally, none of the above-mentioned methods is sufficiently accurate to discriminate between the exo- and endoglycosidic activities of PARG. A number of assays have been designed to specifically monitor the endoglycosidic activity of PARG in protein-bound and protein-free polymer populations, but the most widely used methods are based on the analysis of PAR reaction products on high-resolution DNA sequencing gels^24^ and by HPLC^23,81^. Despite being experimentally challenging, HPLC analysis of PARG degradation products following digestion with snake venom phosphodiesterase (svPDE) remains the method of choice to determine the relative contribution of both PARG glycosidic activities to PAR erasing. In this assay, the exoglycosidic activity allows PARG to attack PAR polymers at the protein-distal chain end to release ADP-ribose units, which are subsequently converted to AMP by cleaving the phophodiester bond with snake venom phosphodiesterase. The endoglycosidic actvity of PARG generates additional chain termini that release supplementary AMP upon double digestion with svPDE, which can be measured to estimate the relative endo/exo activities^23^.

A more recently developed alternative to measure PARG endoglycosidic activity is based on the detection of ADP-ribose oligomers by LC-MS^61^. In this approach, PAR termini are protected with an inactive bacterial PARG^E115Q^ mutant that blocks exoglycosidic cleavage. When human PARG is added to the blocked PAR substrate, only endoglycosidic cleavage can occur. The accumulation of PAR fragments (ADP-ribose oligomers) is detected by LC-MS in the form of specific mass-to-charge ratios and correlated to the endoglycosidic activity of PARG^61^. Although exo- and endoglycosidic mechanisms are essential for efficient dePARylation, it is common to reflect PARG activity in a single value that integrates both activities based on commercially available chemiluminescence- and colorimetric-based detection systems. These assays are suitable for high-throughput screening of PARG inhibitors in addition to antibody-based detection methods^82^. However, immunological detection of PAR is prone to underestimating the presence of residual ADP-ribose oligomers for which the antibodies generally possess low affinity^83^. The recent development of antibody-like MAR- and PAR-binding reagents should prove beneficial to the evaluation of PARG inhibition in cells^84^.

Given the increasingly important role of MAR erasers, a number of approaches have also been developed to facilitate the detection of MAR hydrolase activities. One of the most effective approaches is to use the auto-MARylated PARP-1^E988Q^ mutant as a substrate for MARylation erasers. This PARP-1 mutant is significantly more active than other MARTs and thus represents a robust approach to generate a MARylated substrate. *Bona fide* MARTs such as PARP-10/ARTD10 also have been used as MARylated substrates^49,59,67,68,85^. Furthermore, the oligo(ADP-ribosyl)ated PARP of *H. aurantiacus* was employed as an intermediate length substrate for ADP-ribose erasing assays^66^. Reaction products are generally resolved by SDS-PAGE or TLC. These approaches provide valuable substrate models for ADP-ribosylation erasing studies but may not reflect the diversity and wide range of ADP-ribose polymer species, which could explain the persisting confusion regarding the linkage selectivity of MAR hydrolases. While some MARTs such as PARP-10/ARTD10 appear to be MARylated exclusively on acidic residues^85^, it is less clear which types of ADP-ribose−protein linkages exist in other MARTs and PARP-1^E988Q^. A panel of linkage-specific substrates would be necessary to assess the diversity of MARylation reversal. For example, ADP-ribosylated actin by the arginine-specific ADP-ribosyltransferase CDTa provides a defined substrate for arginine-mediated ADP-ribosylation studies while the threonine-specific transferase TccC3 mono-ADP-ribosylates threonine residues of the same substrate^85^.

It should be kept in mind that the amino-acid sequence surrounding the ADP-ribosylation site is unknown in these substrates and might influence the recognition by the eraser. Similarly, MARylated substrates, such as histones, may carry additional PTM decorations that could also tune the binding affinity of the erasing enzyme. The development of synthetic peptides with site-specific ADP-ribosylations will be particularly useful for dissecting the substrate specificity of ADP-ribose erasers^86^. *Trans*-ADP-ribosylation of synthetic peptides with PARP-1^E988Q^ has been demonstrated by MS analysis but the actual yield of peptide MARylation is probably too low for subsequent biochemical analysis, even after affinity purification^87^. Alternatively, peptide microarrays containing several ADP-ribosylated residues in a variety of sequence contexts may allow profiling of the recognition and processing specificity of MARylation erasers.

## ADP-ribose linkage selectivity of erasers

An intrinsic characteristic of ADP-ribosylation is the molecular heterogeneity and complexity of the reaction product transferred to target substrates. Therefore, ADP-ribosylation needs to be viewed in a length- and site-dependent manner. The site-specific length of PAR polymers is difficult to test experimentally and further studies are needed to characterize the length diversity of PARylated substrates. More progress has been made with regard to determining ADP-ribosylation sites within proteins. The site-specific localization of ADP-ribosylation modifications could be mapped in a system-wide manner in several recent MS-based proteomics studies. Notably, these methods are significantly more challenging and difficult to implement than standard MS-based approaches that only aim for protein identification^56,88–92^.

A survey of the current literature indicates that all chemically reactive amino acids (i.e., excluding those with hydrophobic side chains) may be targeted by ADP-ribosylation under physiological conditions^45,93^. The biological significance of differential ADP-ribosylation site usage is unknown but ADP-ribose−protein linkages appear to be processed by erasers with rigid selectivity (Table 1). For instance, ARH1 hydrolyzes N-linked MARylated arginines^42^, the macrodomain-containing proteins are specific for the carboxyl ester bond formed with the side chains of aspartate and glutamate residues^85^ while ARH3 hydrolyzes O-linked MARylated serines^52^. Although only limited information is available, a modulation of ADP-ribose recognition has been reported for MAR erasers, suggesting that the local amino acid sequence environment influences ADP-ribosylation erasability^52,94^.

The different susceptibility of each type of ADP-ribosylation to degradation by the erasers suggests that the stability of ADP-ribosylation in cells may vary depending on the type of linkages. For example, the absence of a specific enzyme to erase ketoamine-linked ADP-ribose from lysine residues has been hypothesized to be involved in the long-term maintenance of histone epigenetic marks^95^. Furthermore, PAR polymer populations with different half-lives, depending on their length and complexity, have been reported^23,96^. This suggests that recognition and processing of multi-site and multi-structural ADP-ribosylation involves complex coordination of the erasers. However, current atlases of ADP-ribosylation signatures, notwithstanding their importance, from human cancer cells provide little information regarding the occupancy rate of different ADP-ribosylation modifications. At the moment, it is unclear how multiple ADP-ribosylation linkages can be read and transformed into meaningful signalling information.

The termini of a DNA strand break can also be reversibly modified by covalent PARylation in vitro^97–100^, and the ADP-ribose ester bonds of MARylated, phosphorylated double-stranded DNA can be hydrolyzed by MacroD1^101^. PARP-3-mediated MARylation of DNA can also be erased by MacroD2, TARG1, PARG, and ARH3^99^. For DNA MARylation reversal, MacroD1, MacroD2, and TARG1 target the same type of ester bonds (Table 1), while ARH3 activity likely targets the O-glycosidic ribose-ribose bond. Moreover, PARG can efficiently remove MAR moieties attached to DNA phosphate residues - in contrast to its activity on protein substrates^98,99^. This observation emphasizes the importance of exploring the substrate specificity of erasers, which might not be as rigid as initially thought. The role of DNA ADP-ribosylation in the repair mechanisms that maintain the integrity of genomic DNA remains elusive but represents a new dimension in ADP-ribosylation dynamics.

The identification of ADP-ribosylated substrates is undergoing rapid expansion owing to the development of high-sensitivity mass spectrometers. The functional significance of most ADP-ribosylation that occurs on a variety of amino-acid targets is not yet understood. Some of these modifications might be generated nonenzymatically when a biomolecule encounters a reactive metabolite such as ADP-ribose. The formation of ketoamine glycation conjugates on histones lysine and arginine amino acids, in the presence of ADP-ribose, has been documented^102^. This phenomenon might be explained by the local accumulation of ADP-ribose, as a result of PAR hydrolysis by PARG in vitro during sample preparation for MS analysis^56^. However, artefactual glycation by free ADP-ribose released by PARG on lysine and arginine residues was not observed in cell extracts supplemented with free PAR chains and PARG^103^. Alternatively, the accumulation of free ADP-ribose within a confined area might arise from a side reaction based on PARP-1’s abortive NADase activity^104^. Following PARP-1 automodification, NAD^+^ hydrolysis becomes a major component of PARP-1 activity, which releases ADP-ribose that can react with glycation-prone amino groups of proteins or other biomolecules. Therefore, caution is recommended in interpreting results based on the identification of rare, low abundant or atypical ADP-ribosylation modifications.

## A model for cellular PARylation dynamics

The existence of a continuum of ADP-ribose polymer lengths in cells coupled to a variety of amino acid linkages suggests that various erasing modes act together to drive the ADP-ribosylation cycle. PARG possesses the highest level of PAR chain degradation activity. However, its inability to remove the proximal ADP-ribose moiety from proteins illustrates that a complete reversal of ADP-ribosylation likely requires an orchestrated cellular response involving both MAR and PAR erasers^61^. Substrates that would have undergone fast, but partial, trimming of their PARylation modifications by PARG could then be processed by a group of specialized erasers. The rapid initial degradation process likely depends on the synergistic endo/exo dePARylation activity of PARG, considering that endoglycosidic PARG activity also releases protein-bound PAR polymers and predominates in the earliest phases of the degradation process^24^.

**Fig. 4:**
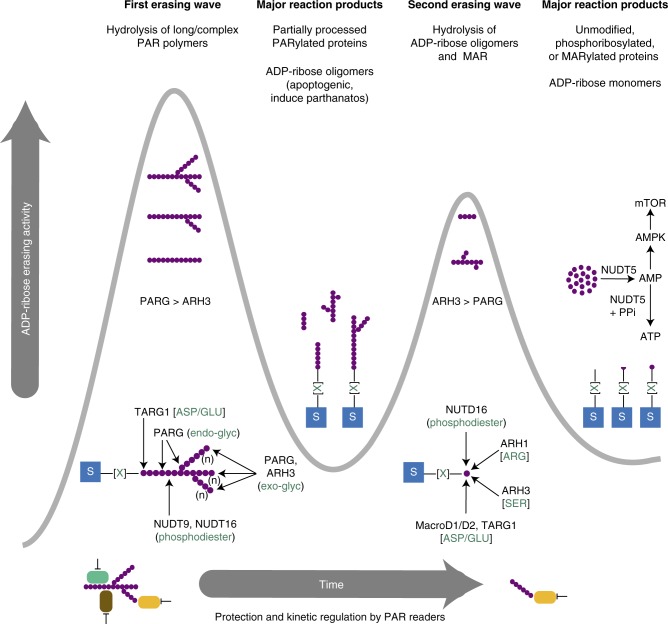
The dynamic mechanism of ADP-ribosylation reversal. ARTDs consume NAD^+^ and transfer ADP-ribose moieties onto target substrates (blue boxes) on different amino acids side chains (green [X]). These proteins can have a variety of different ADP-ribosylation modification patterns, as described in Fig. 1. In the context of severe genotoxic insult, complex PAR polymers composed of large and branched molecules are synthesized by ARTD1 and ARTD2 (PARP-1 and PARP-2). These polymers are rapidly recognized and processed by a variety of erasers in a biphasic mode. At the same time, a variety of PAR readers can bind PAR and regulate the kinetics of the erasing process. In the first phase of the ADP-ribosylation reversal, PARG activity predominates and presumably exceeds ARH3 activity since PARG possesses high affinity for complex polymers and a very rapid and processive exoglycosidic activity toward ribose-ribose linkages. The dePARylation process is enhanced by the unique ability of PARG to cleave in-chain ribose-ribose linkages and branching points owing to its endoglycosidic activity. In addition, TARG1 can contribute to protein dePARylation by detaching entire PAR chains through cleavage of the proximal protein−ribose linkage. As PAR polymers are rapidly shortened by the combined endo- and exoglycosidic activities of PARG, the dePARylation activity drops and partially trimmed apoptogenic ADP-ribose oligomers accumulate. These small PAR fragments induce a second erasing wave in which rate and processivity of PARG is markedly decreased while ARH3 activity becomes dominant. The residual PARG activity and ARH3-catalyzed PAR hydrolysis generate MARylated proteins, which are further degraded by amino acid-specific MAR hydrolases and NUDIX phosphodiesterases. These waves of ADP-ribosylation erasing generate unmodified, phosphoribosylated, and MARylated proteins as well as free ADP-ribose. The latter might be deleterious for the cells and thus recycled by NUDT5 to quickly replenish ATP levels or converted to AMP which activates the AMP kinase (AMPK) and the mTOR signalling pathway

At the same time, the interplay between ADP-ribosylation writers and erasers could regulate the temporal order of the signalling response to PARP-1 activation. For example, the dynamics of ADP-ribosylation reversal strongly depend on the type of PAR polymer synthesized. Large and complex polymers generated during the DNA damage response are short-lived and transient with half-lives of few seconds while the constitutive fraction of PAR is degradation-resistant for hours^96^. Collectively, these observations suggest that ADP-ribosylation erasing may be described as a multistep processing cascade with specific kinetics depending on the physiological context (Fig. 4).

Our understanding of ADP-ribosylation has been substantially advanced by the identification of histone PARylation factor 1 (HPF1) as a regulator of both histone ADP-ribosylation and PARP-1 automodification. This finding demonstrated that a PARP-1-interacting factor can modulate PARP-1’s PARylation activity, switching it to O-linked ADP-ribosylation^105^. In contrast, most previous reports showed that PARP-1 activity is modulated by a variety of DNA lesions^106^, post-translational modifications^107^ or NAD^+^ availability^108^. The observed HPF1-dependent serine-ADP-ribosylation of PARP-1, histones and chromatin-interacting factors as well as accumulating evidence that serine residues are the preferred ADP-ribosylation targets upon DNA damage induction^109–113^ suggest that serine-ADP-ribosylation predominates. This challenges the current model, in which protein ADP-ribosylation is primarily localized to aspartate, glutamate, lysine and arginine residues with cell type- and tissue context-dependent stoichiometries. However, it would be premature to conclude that DNA-dependent PARPs are uniquely engaged in O-glycosidic linkages with serine and, as recently demonstrated, tyrosine residues^114^ in all HPF1-expressing cells and that all other amino acid linkages are in vitro artifacts. For instance, large-scale proteomics studies provided convincing and robust evidence of site-specific glutamate and aspartate ADP-ribosylation^88,115,116^.

As discussed above, HPF1 appears to switch PARP-1 ADP-ribosyltransferase activity toward O-linked ADP-ribosylation. This observation implies that PARP-1-interacting proteins can have profound impact on the PARP-1 enzymatic mechanism. In the case of HPF1, the switch from a carboxyl ester ADP-ribosylation chemistry on acidic glutamate and aspartate amino acids to O-linked ADP-ribosylation of neutral serine residues may be explained by an HPF1-dependent reconfiguration of PARP-1 active site^110,117^. HPF1 accumulates at DNA lesions in a PARP-1-dependent but PAR-independent manner^105^. Considering that PARP-1 relocation to DNA damage sites precedes HPF1 recruitment, PARP-1 could be involved in the glutamate and aspartate ADP-ribosylation of the nucleosomal surface^118^ before switching its catalytic activity toward serine residues as HPF1 accumulates locally. This may indicate a regulatory mechanism with several overlapping waves of linkage-specific ADP-ribosylation (Fig. 4).

The modulation of PARP-1 activity can also be observed in polymer turnover systems that recapitulate PAR metabolism. In these systems, the addition of PARG shifts the ADP-ribose transferase activity of PARP-1 from automodification to histone modification^23,119–121^. Similarly, HPF1 promotes histone ADP-ribosylation and limits PARP-1 hyper-automodification^105^. These results motivate the identification of additional modulators of PARP-1 activity. They also suggest that PARP-1 intramolecular conformational changes may be transmitted via protein-protein interactions. This mechanism is exploited to provide an alternative to common PARP-1 inhibition by antagonizing NAD^+^ binding at the catalytic site^122^. Besides, a network of allosteric communications is known to connect damage recognition to catalytic domain remodelling in order to activate PARP-1^123–127^. The fact that PARP-1 activity depends on protein conformational flexibility is illustrated by the identification of a PARP-1 inhibitor that promotes the formation of a complex specifically through PARP-1 BRCT domain^122^. Although the BRCT domain itself is dispensable for PARP-1 activity^127^, the rigidity of the cross-linked PARP-1 BRCT/small-molecule inhibitor product presumably blocks allosteric communications and the propagation of the activation signal to the active site. We can only speculate how additional effectors might rearrange the PARP-1 active site pocket to enable the formation of alternative ADP-ribosylation linkages. Notwithstanding, different PARP-1 ADP-ribosylation activities within the same pathway further support the notion that cells must produce diverse, specialized erasers.

Non-covalent interactions are thought to play an important regulatory role in ADP-ribose catabolism. For example, a marked inhibition of PARG activity is observed when free PAR is associated with histones or nuclear matrix proteins, most likely through protection of ADP-ribose polymers from PARG-mediated degradation^128^. Alternatively, ADP-ribose polymers that are non-covalently bound to different acceptor proteins may be differentially accessible to PARG^96,129^. Such competition between PAR and MAR-binding proteins (i.e., readers) and erasers adds an additional layer of complexity as PAR readers might influence the kinetics of degradation of polymers and MAR production.

The regulatory model of sequential erasing waves shown in Fig. 4 can be illustrated by the bimodal recruitment kinetics of MacroD2 to sites of laser-induced DNA damage^130^. Following a rapid relocation of MacroD2 in a PARP-1-dependent initial phase, a second slower phase is observed, presumably as a consequence of the accumulation of MARylated species generated through PARG activity. This two-step mechanism reveals that ADP-ribosylation reversal provides a temporal ordering to orchestrate MAR/PAR-regulated pathways. More generally, the different states of ADP-ribosylation and the proteins responding to them may help to sequence and coordinate related reactions and eventually decide cell fate (Fig. 4). Since the loss of the eraser can have consequences other than removing the writer, it is likely that the ADP-ribsolyation intermediates are exploited to activate reactions rather than to simply terminate the actions of the writers.

## Synthetic lethal strategies with PARG inhibitors

Fueled by the success of PARP inhibition (PARPi) as a therapeutic strategy for the treatment of many cancers, the field is now exploring the therapeutic potential of PARG inhibition (PARGi). Probably inspired by the postulated nucleic acid-like helical conformation of PAR^131^, Tavassoli and colleagues reported that DNA intercalators can form a complex with PAR and protect it from PARG hydrolysis^132^. These homopolynucleotide intercalators (e.g., ethacridine) were the first class of compounds used to inhibit PARG. However, the influence of DNA intercalators on PARG activity is primarily indirect by restricting access of substrates rather than through direct interaction with PARG. Later, naturally occurring polyphenolic compounds such as the tannins were found to inhibit PARG activity^133^. In particular, Gallotannin^134^ was shown to inhibit PARG and to be synthetic lethal to BRCA2-deficient tumors^135^. However, the utility of this compound was subsequently called into question as it exhibits nonspecific effects and is essentially cell membrane impermeable^79^.

Despite its lack of cell permeability, one of the most widely used and best characterized PARG inhibitors is adenosine diphosphate hydroxymethyl pyrrolidinediol (ADP-HPD)^136,137^. Photoincorporation studies with analogues of ADP-HPD showed that the high molecular weight and branched PAR bind PARG at a different sites than short, linear polymers and ADP-HPD^138^. This is consistent with the identification of a secondary substrate binding site on PARG, hypothesized to be involved in its processive behavior^77^. These results indicate that small-molecule inhibitors of PARG might have different effects on PAR processing by modulating its ratio of exo/endoglycosidic activities.

To overcome cellular permeability issues with PARG inhibitors, a new generation of compounds was developed by Hergenrother and colleagues^78^. Rhodamine-based PARG inhibitors (RBPIs) proved to be potent and selective PARG inhibitors since they do not inhibit ARH3 as does ADP-HPD^78^. However, these compounds exhibited only low micromolar inhibitory activity against PARG. Other non-tannin inhibitors such as the GPI 1552 were reported to protect against neuronal damage^139^ and potentiate temolozomide anti-metastatic activity in brain tumours^140^. A careful reexamination of the actual evidence for PARG inhibition leads to the conclusion that GPI 1552 was inadequate for a pharmacological evaluation of PARG^79^.

More recently, using the cell permeable PARGi PDD00017273^141^, Bryant and colleagues demonstrated that PARGi treatment selectively kills BRCA1-, BRCA2-, PALB2-, FAM175A/ABRAXAS-, and BARD1-depleted cells in the absence of any exogenous DNA damaging agents. The underlying mechanism for this synthetic lethality is that PARGi provokes replication forks stalling and a reduction of DNA double-strand break repair via homologous recombination (HR). An alternative explanation is that inhibition of PARG might cause irreversible PAR association with several proteins needed to complete HR^142^. Importantly, PARGi does not phenocopy PARPi. PARGi induces a rapid increase in IR-induced activation of DNA-PK and impairs normal mitotic progression. This suggests that PARG has different effects on activation of DNA damage repair pathways following ionizing radiation, consistent with the notion that blocking PAR removal has a different consequence to inhibiting PAR addition^143^.

Genetic studies have suggested PARG inhibitors as chemosensitizing agents. PARG-deficient cells display centrosome amplification and accumulate aberrant mitotic figures, which induced either polyploidy or cell death by mitotic catastrophe^144^. ES cells derived from knock-out PARG mice showed enhanced sensitivity towards γ-irradiation and other forms of ionizing radiation^145^. More recently it was shown that PARG suppression potentiates the toxicity of radiation therapy in BRCA-deficient cells^142^ and that PDD00017273 radiosensitizes MCF-7 cells^143^.

PARG protein expression can be regulated by the stabilization of its mRNA by the RNA-binding protein HuR^146^. In pancreatic ductal adenocarcinomas (PDA) cells, genetic deletion of HuR enhances PARPi sensitivity. In this context, the PARPi-induced toxicity is attributable to downregulation of PARG expression. The inhibition of HuR can also re-sensitize PDA cells to PARPi, suggesting that the loss of PARG activity could enhance the clinical effectiveness of PARP inhibitors^146^. In contrast, Gogola and colleagues have shown that PARPi-resistance can be mediated by PARG downregulation^147^. The loss of PARG activity in BRCA2-deficient tumours treated with potent PARP-1 inhibitors is sufficient to restore PAR formation and rescue PARP-1 downstream signaling. PARG depletion indeed occurs in triple-negative breast cancers and serous ovarian cancers. When treatment-naive TNBC biopsies from women eligible for PARPi treatment were analyzed for PARG expression, lack of PARG occurred in some areas of the tumours^147^. This suggests that these tumour sections can become de facto resistant to PARPi treatment. Further studies are needed to clarify the role of PARG and the accumulation of PAR polymers that survived erasing in a dynamic system that has undergone profound alterations. Differences in genetic backgrounds can certainly account for contradictory results (e.g., DNA damage response-proficient vs -deficient cells) but the nature of PAR itself (either free or protein-bound) might also be an important and significant clue to interpret these results. Although there is a bright future for PARG inhibitors, so far they are only effective at relatively high doses in contrast to PARP-1 inhibitors characterized with half maximal inhibitory concentration in the low nanomolar range. In addition, the fact that PARG activity can be regulated at multiple levels with respect to PAR length and branching patterns poses a particular challenge.

## Concluding remarks and future challenges

In this review, we have provided an overview of the dynamic and reversible processes that regulate ADP-ribosylation. Determining the contribution of each regulator in this delicate equilibrium represents a daunting challenge. Experimental dissection of these processes are complicated by the heterogeneous nature of ADP-ribosylation, which needs to be addressed by the development of specialized analytical methods.

Substantial progress has been made to understand the mechanisms that contribute to ADP-ribosylation reversal, yet several obstacles need to be overcome: (1) Sensitive and reproducible methods to monitor physiological MAR and PAR levels in cells are difficult to implement; (2) better methods are needed to evaluate site-specific PAR chain length distribution in ADP-ribosylated substrates; (3) measuring kinetics and performance of erasers are precluded by the lack of standardized and defined substrates; (4) the ADP-ribosylation conjugation chemistry and linkage selectivity of erasers need to be further clarified; (5) enzyme-specific targets and interactors, especially for the MARTs and ARHs, remain largely unknown; (6) the modulatory effect of many ADP-ribose readers, which have multiple binding sites for the same ligand is largely unresolved; (7) the biological relevance of site-specific ADP-ribosylation events is often difficult to determine.

Despite these obstacles and intricacies, the machinery responsible for the processing of ADP-ribose is now beginning to be revealed. A more detailed understanding of the interplay between ADP-ribosylation writers, readers and erasers, at a molecular level, will be required to correlate these dynamics with cellular responses and translate them into clinical applications.
